# Hemodynamic Changes Following Landiolol Initiation in Patients With Critical Illness Who Developed Tachycardia During Dobutamine Infusion: A Retrospective Observational Study

**DOI:** 10.7759/cureus.92273

**Published:** 2025-09-14

**Authors:** Daichi Fujimoto, Satoshi Mizobuchi, Norihiko Obata

**Affiliations:** 1 Department of Anesthesiology, Kobe University Hospital, Kobe, JPN; 2 Department of Anesthesiology, Sanda City Hospital, Sanda, JPN

**Keywords:** dobutamine, hemodynamic changes, intensive care medicine, landiolol, mean arterial pressure, tachycardia

## Abstract

Background and objective

Dobutamine, a β1-adrenergic agonist, is frequently used to enhance myocardial contractility in patients with critical illness. However, it also increases tachyarrhythmia and myocardial oxygen consumption. Landiolol, an ultra-short-acting and highly β1-selective β-blocker, is used to manage tachycardia in patients with critical illness; however, the combination of a β-agonist and β-blocker seems contradictory at first glance. Previous studies have examined this combination in healthy subjects; however, its effects on patients with critical illness have not been fully elucidated. Hence, this study aimed to address that gap in the literature.

Methods

This single-center, retrospective, observational study included adult ICU patients who received combination therapy with dobutamine and landiolol between January 1, 2014, and December 31, 2023. Patients who received combination therapy for less than 24 hours or who required extracorporeal circulatory support were excluded. Continuous hemodynamic data were extracted from electronic medical records. Primary outcomes were changes in heart rate (HR) and mean arterial pressure (MAP) after initiation of landiolol administration. Secondary outcomes were achievement of HR <110 bpm, incidence of hypotension (MAP <65 mmHg), change in lactate levels, and rhythm conversion in patients with atrial fibrillation (AF). Linear mixed-effects models were used to evaluate changes in HR and MAP after the initiation of landiolol treatment.

Results

A total of 72 patients were included in the final analysis. Landiolol treatment was significantly associated with a significant decrease in HR (β = −2.61, p < 0.001) without a significant decrease in MAP. Landiolol treatment also significantly decreased lactate levels (p = 0.029). In patients with atrial fibrillation (n = 53), 79.2 % converted to sinus rhythm or paced rhythm after the initiation of landiolol treatment.

Conclusions

Landiolol effectively reduced HR without reducing MAP in critically ill patients with dobutamine-induced tachycardia. Furthermore, landiolol may promote rhythm control and improve metabolic parameters such as lactate levels.

## Introduction

Dobutamine, a β-adrenergic receptor agonist, mainly acts on β1 receptors to improve myocardial contractility. It is frequently used for treating acute heart failure and cardiogenic shock and is typically administered in patients with critical illness. Although dobutamine improves cardiac contractility, it also increases myocardial oxygen consumption, thereby elevating the risk of myocardial ischemia and tachyarrhythmia [1]. Dobutamine use in patients with sepsis and those undergoing cardiac surgery has been reported to induce arrhythmias [2,3]. When tachycardia develops, the administration of landiolol, a β-adrenergic receptor blocker, is sometimes considered. Landiolol is regarded as an ideal agent for heart rate (HR) control owing to its high β1-selectivity (β1/β2 ratio of 255:1), potent negative chronotropic effect, minimal negative inotropic effect, and extremely short elimination half-life of approximately 4 min [4].

In patients with critical illness, including those following cardiac surgery or with sepsis, concomitant use of β-adrenergic agonists and β-blockers is occasionally observed. This approach presents a pharmacological paradox as it involves simultaneous stimulation and inhibition of the same adrenergic receptor subtype (β1). The theoretical concern is that co-administration may cause opposing hemodynamic actions, potentially leading to instability or attenuation of therapeutic benefits. Tachycardia can be deleterious by reducing diastolic filling time and increasing myocardial oxygen demand [5], which landiolol may help suppress to improve hemodynamics. However, the safety and hemodynamic effects of this pharmacological conflict remain poorly elucidated. To date, only a few studies by Krumpl et al. have investigated the pharmacological effects of short-term landiolol administration during dobutamine infusion in healthy adult volunteers [6,7]. Notably, no studies have evaluated the combined use of dobutamine and landiolol in patients with critical illness. Therefore, we retrospectively collected hemodynamic data from patients admitted to our intensive care unit (ICU) and treated with dobutamine and landiolol to investigate the effects of this combination.

Part of this study was presented at the 67th Annual Meeting of the Japanese Society of Anesthesiologists, Kobe, Japan, in 2020.

## Materials and methods

Study design and ethical considerations

This was a single-center retrospective study. The Kobe University Ethics Committee approved this study without the requirement for informed consent from the patients (approval number: B240187; approval date: December 20, 2024).

Participants

We included patients who were admitted to the ICU of our hospital from January 1, 2014, to December 31, 2023, and who received landiolol combined with dobutamine. The exclusion criteria were as follows: (1) patients who received combined dobutamine and landiolol therapy for <24 h; (2) those who underwent extracorporeal membrane oxygenation (ECMO), an IMPELLA® device, or a ventricular assist device (VAD); and (3) those with missing vital sign data.

**Figure 1:**
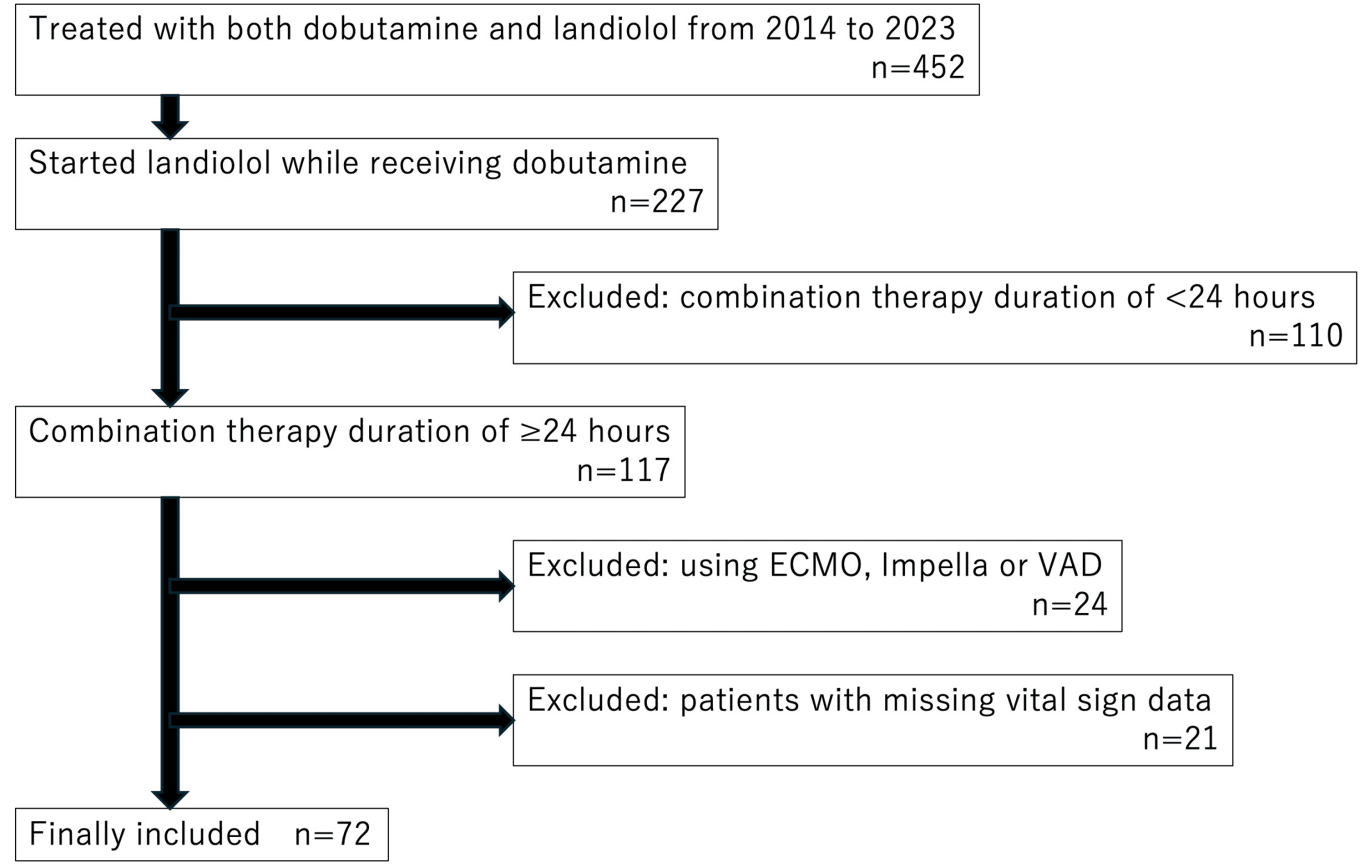
Flowchart depicting patient selection ECMO: extracorporeal membrane oxygenation; VAD: ventricular assist device

From 2014 to 2023, 452 patients were admitted to our ICU and were treated with a combination of dobutamine and landiolol. Among these patients, 227 who initiated concomitant landiolol administration during dobutamine therapy were identified. Of these patients, 110 who received concomitant therapy for <24 h were excluded. From the remaining 117 patients, 24 who required ECMO, IMPELLA® device, or VAD were also excluded. Additionally, 21 patients were excluded owing to missing vital sign data. Ultimately, 72 patients were included in the final analysis (Figure 1).

Collected data

The following baseline patient characteristics were collected: sex, age, height, body weight, body mass index, Sequential Organ Failure Assessment (SOFA) score [8] at ICU admission, length of ICU stay, ICU mortality, medical history, reason for dobutamine and landiolol administration, and patient status during the study period, including intubation status, use of continuous hemodiafiltration (CHDF), and intra-aortic balloon pumping (IABP).

In addition, we recorded circulation-related medications administered during the study period(dobutamine [1], noradrenaline [9], dopamine [10], remifentanil [11], fentanyl [12], propofol [13], and dexmedetomidine [14]), use of antiarrhythmic drugs, defibrillation, or cardioversion, as well as the following clinical data at the time of observation: lactate level and SOFA score.

During the ICU stay, continuous minute-by-minute blood pressure and HR data were acquired from the electronic medical record system (PrimeGAIA, Nihon Kohden, Tokyo, Japan). When artifacts or missing values were noted in blood pressure or HR data, the following adjustments were made before statistical analysis. (1) Blood pressure values that could be considered artifacts were defined as the difference between systolic and diastolic blood pressures of ≤5 mmHg and were treated as missing data. (2) Missing HR data were imputed using the pulse rate data from a SpO₂ monitor. Data were considered missing when neither HR nor pulse rate data were available. (3) When missing data occurred within 10 minutes, the missing value was replaced with the average of the nearest available data points before and after the missing interval. (4) When missing data occurred on only one side (either before or after), the missing value was replaced with the nearest available data point. (5) Cases with missing data for >10 min were excluded from the analysis.

Outcomes

Primary Outcome

The change in HR and mean arterial pressure (MAP) after landiolol administration initiation was the primary outcome.

Secondary Outcomes 

Secondary outcomes included (1) achievement of HR < 110 bpm, (2) incidence of MAP < 65 mmHg, (3) changes in blood lactate levels, and (4), in a subgroup of patients with atrial fibrillation (AF) as an indication for landiolol, the conversion rate from AF to sinus rhythm or pacing rhythm was evaluated.

Sensitivity Analyses

To assess the robustness of the primary outcome, AF history, SOFA score, and age were individually added to the primary model, and model fit was evaluated using the Akaike Information Criterion (AIC) [15].

Sample size calculation and statistical analysis

A total of 72 patients were included in the statistical analyses. As this was a retrospective observational study conducted for exploratory purposes, no formal sample size calculation was performed. The study findings are based on real-world evidence data from our ICU. Categorical variables were presented as numbers (percentages), and continuous variables were expressed as medians with interquartile ranges (IQRs).

For the primary analysis, a linear mixed-effects model was employed for evaluating changes in HR and MAP following landiolol initiation. The fixed effects included the landiolol dosage. Potential confounders affecting hemodynamics (dobutamine [1], dexmedetomidine [14], fentanyl [12], propofol [13], and noradrenaline [9]) were included as fixed effects. Patient identification number (ID) was treated as a random effect to account for the repeated measures. The effect estimates (β coefficients) and 95% confidence intervals were derived from the fixed effects in the mixed-effects models. Models were fitted using restricted maximum likelihood, and p-values were computed using Satterthwaite’s approximation.

In addition, as a supplementary analysis of temporal trends, hourly HR and MAP values within 24 h following landiolol initiation were analyzed using the Friedman test, with Dunn’s test used for post-hoc comparisons when appropriate. Mixed-effects logistic regression models were used for binary secondary outcomes. Lactate levels were analyzed using a linear mixed-effects model with the same fixed and random effects as the primary model.

Statistical analyses were performed using R (Version 4.4.1; R Core Team, Vienna, Austria) and SigmaPlot (Version 16; Grafiti LLC, California). p < 0.05 was considered statistically significant.

## Results

Patient characteristics

The patient characteristics are shown in Table 1. Of the 72 patients, 48 (66.7%) were male, with a median age of 76.5 (IQR, 67-82) years. The median SOFA score at ICU admission was 11 (IQR, 8.5-14), and the median ICU length of stay was 13.5 (IQR, 6-22) days. Hypertension was the most common comorbidity (26 patients, 36%), followed by chronic kidney disease (24 patients, 33.3%), and diabetes mellitus (23 patients, 31.9%). Postoperative valvular heart disease was the most common primary diagnosis (23 patients, 32%), followed by coronary revascularization (12 patients, 16.7%) and septic shock (8 patients, 11.1%). AF was the most common reason for initiating landiolol during dobutamine use (54 patients, 75%), followed by sinus tachycardia (14 patients, 19.4%). Intubation was required in 48 patients (66.7%), CHDF was performed in 17 patients (23.6%), and IABP was used in 6 patients (8.3%).

**Table 1:** Baseline characteristics BMI: body mass index; ICU: intensive care unit; CHDF: continuous hemodiafiltration; IABP: intra-aortic balloon pumping; SOFA: Sequential Organ Failure Assessment

Variable	Number (%)	Median (quantile)
Basic data		
Sex, male	48 (66.7)	
Age (year)		76.5 (67–82)
Height (cm)		162.3 (152.8–168.3)
Body weight (kg)		55.8 (48.4–63.2)
BMI (kg/m^2^)		21.8 (19.5–25.0)
SOFA score at ICU admission		11 (8.5–14)
Length of ICU stay (days)		13.5 (6–22)
Mortality during ICU stay	12 (16.0)	
Comorbidities		
Hypertension	26 (36.1)	
Chronic kidney disease	24 (33.3)	
Dialysis	8 (11.1)	
Diabetes	23 (31.9)	
Ischemic heart disease	23 (31.9)	
Chronic atrial fibrillation	17 (23.6)	
Heart failure	12 (16.7)	
Pacemaker user	5 (6.9)	
Reason for using dobutamine		
Postvalvular surgery	23 (32.0)	
Coronary revascularization		
Coronary artery bypass grafting	9 (12.5)	
Percutaneous coronary intervention	3 (4.2)	
Septic shock	8 (11.1)	
Great vessel surgery	7 (9.7)	
Respiratory failure	7 (9.7)	
Heart failure	4 (5.6)	
Post-resuscitation myocardial injury	4 (5.6)	
Left ventricular rupture	2 (2.8)	
others	5 (6.9)	
Reason for using landiolol		
Atrial fibrillation	54 (75.0)	
Sinus tachycardia	14 (19.4)	
Premature ventricular contraction	1 (1.4)	
Atrial tachycardia	1 (1.4)	
Cytokines	1 (1.4)	
Unknown	1 (1.4)	
Status of patients during the study period		
Intubation	48 (66.7)	
CHDF use	17 (23.6)	
IABP use	6 (8.3)	

Circulation-related medications

Circulation-related Medications used during this study period are shown in Table 2. The median dose of landiolol was 2.1 (IQR, 1.7-3.5) μg/kg/min, and the median dose of dobutamine was 3.3 (IQR, 2.1-4.2) μg/kg/min. Among catecholamines other than dobutamine, noradrenaline was administered to 49 patients (68%), and dopamine was administered to 7 patients (9.7%). The median dose of noradrenaline was 0.07 (IQR, 0.05-0.18) μg/kg/min, and the median dose of dopamine was 6.6 (IQR, 4.9-7.8) μg/kg/min. Regarding analgesics, remifentanil and fentanyl were used in 1 (1.4%) and 51 (70.1%) patients, respectively. The median dose of fentanyl was 0.82 (IQR, 0.48-1.04) μg/kg/h. Propofol and dexmedetomidine were administered as sedatives to 29 (40.3 %) and 28 (38.9 %) patients, respectively. The median doses of propofol and dexmedetomidine were 0.65 (IQR, 0.43-1.08) mg/kg/h and 0.38 (IQR, 0.34-0.45) μg/kg/h, respectively.

**Table 2:** Medications administered during the study period

Variable	Number (%)	Dose
Landiolol (μg/kg/min)	72 (100)	2.1 (1.7–3.5)
Dobutamine (μg/kg/min)	72 (100)	3.3 (2.1–4.2)
Noradrenaline (μg/kg/min)	49 (68)	0.07 (0.05–0.18)
Dopamine (μg/kg/min)	7 (9.7)	6.6 (4.9–7.8)
Remifentanil (μg/kg/min)	1 (1.4)	0.056
Fentanyl (μg/kg/h)	51 (70.1)	0.82 (0.48–1.04)
Propofol (mg/kg/h)	29 (40.3)	0.65 (0.43–1.08)
Dexmedetomidine (μg/kg/h)	28 (38.9)	0.38 (0.34–0.45)

Outcomes

Heart Rate

We analyzed the association between HR and landiolol administration using linear mixed-effects models, adjusted for dobutamine, dexmedetomidine, fentanyl, propofol, and noradrenaline, and included patient ID as a random intercept. The baseline HR was approximately 102.4 bpm. Landiolol dosage was significantly associated with HR reduction (β = −2.6, p < 0.001), similar to fentanyl administration (β = -7.7, p < 0.001). In contrast, the dobutamine dosage was significantly associated with increased HR (β = 2.9, p < 0.001). Dexmedetomidine, propofol, and noradrenaline effects were not significant (Table 3).

**Table 3:** Results of linear mixed-effects model for heart rate changes following landiolol administration P-values were derived from the linear mixed-effects model using Satterthwaite’s approximation

Variable	Unadjusted		Adjusted		
	Effect estimate (95% CI)	p-value	Effect estimate (95% CI)	p-value	
Landiolol	−3.2 (−3.9 to −2.5)	<2 × 10^−16^	−2.6 (−3.6 to −1.9)	3.6 × 10^−12^	
Dobutamine	3.5 (2.6 to 4.5)	1.7 × 10^−12^	2.9 (2.0 to 3.9)	6.9 × 10^−9^	
Dexmedetomidine	−12.5 (−21.3 to −3.7)	0.0053	−6.7 (−15.7 to 2.2)	0.14	
Fentanyl	−7.7 (−11.7 to −3.8)	0.00012	−7.7 (−11.7 to −3.8)	0.00015	
Propofol	−0.6 (−3.9 to 2.7)	0.72	0.4 (−2.9 to 3.8)	0.8	
Noradrenaline	−8.3 (−20.9 to 4.3)	0.2	−10.8 (−23.2 to 1.7)	0.09	

**Figure 2:**
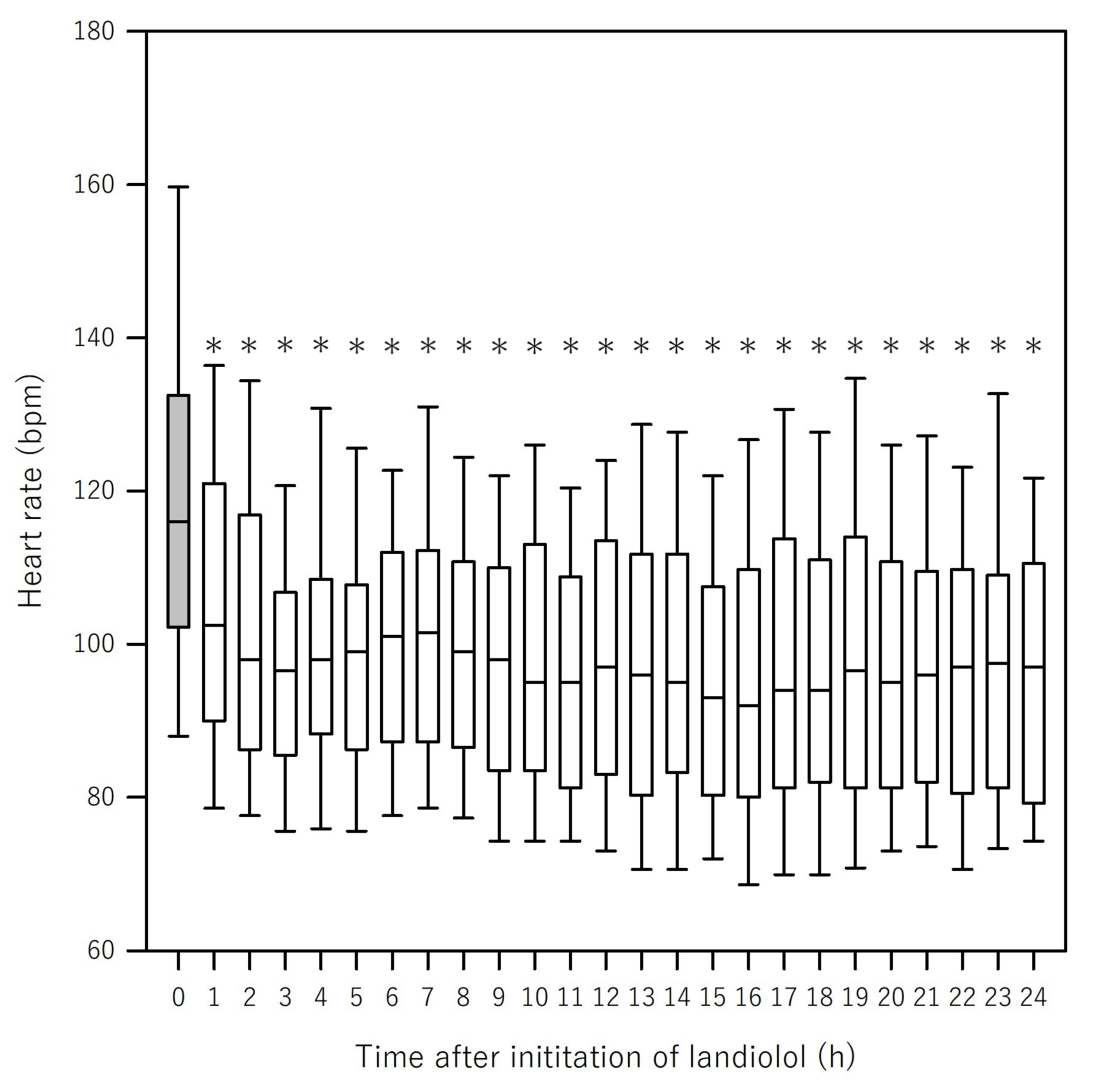
Change of heart rate after initiation of landiolol (Unadjusted) Figure 2 shows a significant reduction in heart rate (HR) after the initiation of landiolol (Friedman test: χ²(24) = 155.6, p < 0.001). Post hoc analysis using Dunn’s test revealed that the HR was significantly lower at all observation time points than at time 0. An asterisk (*) indicates statistical significance (p < 0.05)

Supplementary analyses incorporating covariates, such as age, SOFA score, and AF, consistently demonstrated that landiolol administration was significantly associated with decreased HR (all p < 0.001) (Table 4, left panel). In these sensitivity analyses, AIC values were also evaluated to assess the model fit, which supported the robustness of the association. Figure 2 and Table 8 (Appendices) illustrate a significant reduction in HR following landiolol initiation (Friedman test χ² (24) = 155.6, p < 0.001), with HR being significantly lower at all observation times, particularly from 1 h onward.

**Table 4:** Effects of landiolol on heart rate in linear mixed-effects models with stepwise inclusion of confounding covariates (fixed effects) AF: atrial fibrillation; AIC: Akaike Information Criterion; HR: heart rate; MAP: mean arterial pressure; SOFA: Sequential Organ Failure Assessment

Variable	HR			MAP		
	Effect estimate (95% CI)	p-value	AIC	Effect estimate (95% CI)	p-value	AIC
Main model	−2.6 (−3.4 to −1.9)	3.6 × 10^−12^	14,808.64	0.02 (−0.55 to 0.585)	0.96	13,994.25
+Age	−2.6 (−3.3 to −1.9)	5.7 × 10^−12^	14,810.90	0.01 (−0.56 to 0.579)	0.98	13,998.60
+SOFA	−2.6 (−3.3 to −1.8)	1.2 × 10^−11^	14,616.87	0.03 (−0.54 to 0.60)	0.92	13,816.63
+AF history	−2.6 (−3.3 to −1.9)	4.8 × 10^−12^	14,803.87	0.01 (−0.56 to 0.58)	0.97	13,992.04

Mean Arterial Pressure

Linear mixed-effects models were employed for evaluating the MAP changes with landiolol dosage, adjusting for the same covariates and random intercepts. The baseline MAP was approximately 74.7 mmHg. Propofol was associated with a modest reduction in MAP (β = -3.72, p = 0.006). Other variables, including landiolol, dexmedetomidine, fentanyl, and noradrenaline, were not statistically significant (Table 5).

**Table 5:** Results of linear mixed-effects model for mean arterial pressure changes following landiolol administration P-values were derived from the linear mixed-effects model using Satterthwaite’s approximation

Variable	Unadjusted		Adjusted	
	Effect estimate (95% CI)	p-value	Effect estimate (95% CI)	p-value
Landiolol	0.10 (−0.45 to 0.66)	0.71	0.02 (−0.55 to 0.59)	0.96
Dobutamine	−0.75 (−1.5 to −0.014)	0.046	−0.67 (−1.4 to 0.09)	0.084
Dexmedetomidine	−4.6 (−11.0 to 2.1)	0.18	−1.9 (−8.7 to 4.9)	0.59
Fentanyl	−3.1 (−5.9 to −0.19)	0.037	−2.0 (−5.0 to 0.98)	0.19
Propofol	−4.2 (−6.7 to −1.7)	0.72	−3.7 (−6.3 to −1.1)	0.0057
Noradrenaline	1.2 (−8.2 to 10.5)	0.81	2.3 (−7.1 to 11.8)	0.63

**Figure 3:**
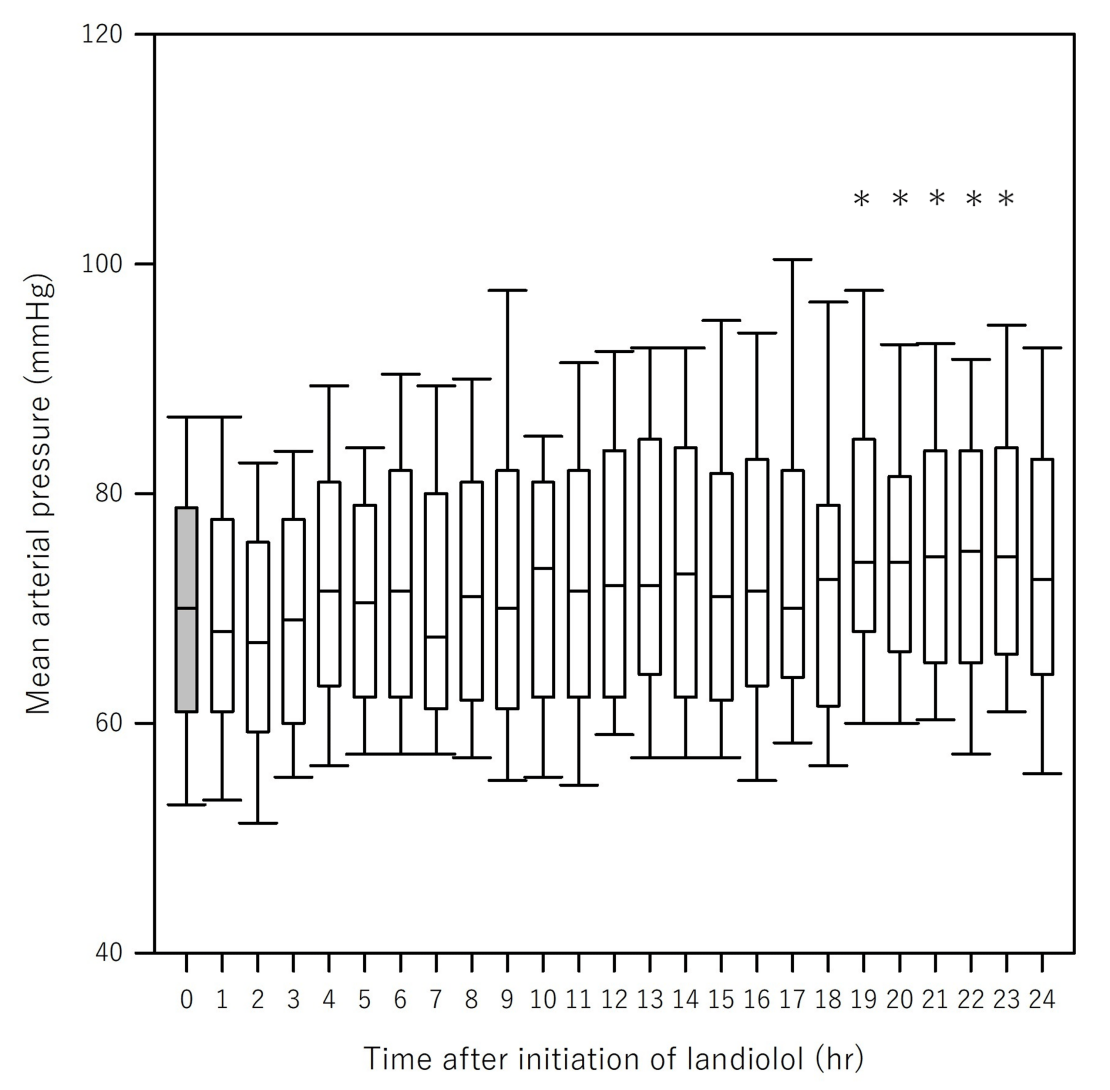
Change of mean arterial pressure after initiation of landiolol (Unadjusted) Figure 3 shows the changes in mean arterial pressure (MAP) after landiolol initiation. An overall increase in MAP was observed following landiolol administration (Friedman test: χ²(24) = 101.3, p < 0.001). Post hoc analysis using Dunn’s test revealed that MAP was significantly higher between 19 and 23 h compared to time 0. An asterisk (*) indicates statistical significance (p < 0.05)

Supplementary analyses incorporating covariates, including age, SOFA score, and AF, consistently demonstrated that landiolol administration was not significantly associated with a reduction in MAP (Table 4, right panel). Moreover, AIC values were calculated in these models and were consistent with the absence of a significant association between landiolol and MAP. Figure 3 and Table 9 (Appendices) show an overall increase in MAP following landiolol administration (Friedman test χ² (24) = 101.3, p < 0.001). MAP significantly increased from 19 to 23 h.

Secondary Outcomes

Mixed-effects logistic regression revealed that higher landiolol doses (β = 0.29, p < 0.001) significantly increased the odds of achieving an HR of <110 bpm. Landiolol dosage alone was not associated with the risk of hypotension (β = −0.06, p = 0.41) but was associated with lower lactate levels (β = -0.07, p = 0.029) (Table 6). Of the 53 patients (73.6%) diagnosed with AF, 42 (79.2%) achieved conversion to a sinus or paced rhythm, with a median time of 2.8 hours (IQR, 1.4-8.4 hours). Additional AF treatments encompassed amiodarone (8.3%) and defibrillation or cardioversion (6.9%) (Table 7).

**Table 6:** Effects of landiolol on secondary outcomes in mixed-effects models P-values were derived from mixed-effects models (logistic regression for HR and MAP, linear regression for lactate) HR: heart rate; MAP: mean arterial pressure

Variable	Unadjusted		Adjusted	
	Effect estimate (95% CI)	p-value	Effect estimate (95% CI)	p-value
HR	0.31 (0.16–0.45)	0.000026	0.29 (0.14–0.43)	0.00015
MAP	−0.0025 (−0.13 to 0.12)	0.97	−0.06 (−0.19 to 0.077)	0.41
Lactate	−0.07 (−0.13 to −0.014)	0.016	−0.07 (−0.13 to −0.007)	0.029

**Table 7:** Medications for AF treatment AF: atrial fibrillation

Variable	Number (%)
Amiodarone	6 (8.3)
Procainamide	4 (5.6)
Verapamil	1 (1.3)
Pilsicainide	0 (0)
Defibrillation or cardioversion	5 (6.9)

## Discussion

Main results

This retrospective observational study investigated hemodynamic changes following landiolol treatment initiation in patients with critical illness who developed tachycardia during dobutamine infusion. The main findings demonstrated that landiolol administration was significantly associated with decreased HR, without a corresponding decrease in MAP. Furthermore, landiolol use was significantly associated with a higher achievement rate (HR < 110 bpm) and a reduction in blood lactate levels. Furthermore, in patients who received landiolol for AF, approximately 80% converted to sinus rhythm or paced rhythm, suggesting the effectiveness of landiolol in achieving rhythm control. The combined use of β-adrenergic agonists (dobutamine) and β-blockers (landiolol) in patients with critical illness with conditions, such as heart failure or sepsis, is theoretically considered contradictory owing to their opposing pharmacological effects. However, this study suggests the potential clinical utility of such a combination.

Comparison with previous reports

Studies on the combined administration of dobutamine and landiolol are limited to the series of studies by Krumpl et al. in healthy adults [6,7]. The study involved 16 healthy volunteers in whom tachycardia was induced by dobutamine, followed by landiolol administration. The results demonstrated that the effect appeared within 16 min following landiolol initiation, with the HR gradually decreasing to a steady state. Dobutamine administration caused a rapid increase in blood pressure; however, the combination with landiolol resulted in decreased blood pressure [7]. Furthermore, landiolol more effectively reduced the dobutamine-induced tachycardia and hypertension than esmolol [6].

Similarly, in the present study, HR was significantly reduced following the addition of landiolol compared with that before combination therapy. However, no decrease in blood pressure was noted in our patients. This discrepancy may be explained by the difference in dosing; the median landiolol dose in our study was approximately 2 μg/kg/min, whereas Krumpl et al. used higher doses (up to 10 μg/kg/min). The higher dose in their studies likely contributed to the observed blood pressure reduction following landiolol initiation.

Interpretation

There are several interpretations of this study’s findings. First, although the HR decreased with the concomitant use of dobutamine and landiolol, MAP was maintained. Previous research on landiolol in postoperative AF has indicated that careful titration of low-dose landiolol effectively controls tachycardia without significantly decreasing blood pressure [16]. In patients with tachycardia-associated AF, landiolol demonstrated a strong negative chronotropic effect and a weak negative inotropic effect. Compared with esmolol, landiolol has less impact on blood pressure and is considered safe even in hemodynamically unstable patients [17]. Experimental studies further support these observations. Sugiyama et al. demonstrated that ONO-1101, an experimental form of landiolol, produced significant HR reduction while only slightly affecting myocardial contractility in an in vivo canine model, compared with esmolol [18]. These findings suggest that, even at low doses, landiolol exerts sufficient negative chronotropic effect while minimizing inotropic suppression. In our study, the linear mixed-effects model confirmed that landiolol administration was significantly associated with reduced HR but not with decreased MAP. Therefore, consistent with its effects in AF [16,17], landiolol was also effective against dobutamine-induced tachycardia, facilitating HR reduction and blood pressure preservation. The maintenance of MAP despite HR reduction may also be partially attributed to dobutamine-induced residual adrenergic stimulation.

Second, decreased blood lactate levels were observed following landiolol administration. Previous studies have reported that ultra-short-acting β1-blockers improved 72 h lactate clearance in patients with sepsis [19]. Although this finding supports our results, it should be noted that lactate clearance is a complex and dynamic process that involves production and metabolism [20]. Therefore, the direct causal relationship between landiolol administration and lactate level reduction remains unclear.

Third, regarding AF, several previous studies have demonstrated the efficacy of landiolol in controlling AF. In this study, 53 patients (73.6%) with dobutamine-induced tachycardia developed AF, which was successfully controlled by landiolol in 42 patients (79.2%). This effect is believed to be due to the ability of landiolol to slow conduction through the sinoatrial and atrioventricular nodes, as well as inhibit ectopic conduction pathways, which contributes to its effectiveness in AF management [21].

Strengths and limitations

The use of continuous minute-by-minute vital sign data, enabling a comprehensive evaluation of hemodynamic changes using mixed-effects modeling, was a major strength of this study. Adjusting for multiple confounding factors and implementing sensitivity analyses further strengthened the robustness of the findings.

However, this study had several limitations. First, this was a single-center retrospective study with a limited sample size, making it challenging to establish causal relationships. Second, although tachycardia was attributed to dobutamine, other potential causes, including sepsis, postoperative inflammatory response, and dopamine [10] (not included in the analysis owing to the small number of patients who used it), may have contributed, and we could not clearly isolate dobutamine as the sole cause. Third, this study was limited to ICU patients who were administered landiolol; therefore, selection bias may be present owing to the attending physicians’ clinical judgment. In addition, the dosing strategies for both landiolol and dobutamine were determined at the discretion of the attending physicians, which may affect the reproducibility of our findings. Fourth, cardiac function during dobutamine initiation or following treatment was not evaluated; therefore, the potential improvement in cardiac performance from the combined therapy remains unknown. Finally, the timing and frequency of lactate level changes may vary across patients.

To validate these findings, future prospective studies with standardized administration protocols are warranted. Incorporating cardiac functional assessments, including echocardiography, would further help identify appropriate patient populations and optimize dosing strategies.

## Conclusions

In patients receiving dobutamine who develop tachycardia, the concomitant use of landiolol effectively reduced HR without lowering blood pressure. Moreover, landiolol administration was associated with a higher likelihood of achieving the target HR (<110 bpm) and with decreased blood lactate levels, suggesting an improvement in tissue perfusion. These findings indicate that landiolol may provide hemodynamic stabilization in this high-risk population. Future prospective studies are warranted to clarify whether these favorable physiological effects translate into improved clinical outcomes, particularly regarding organ dysfunction and survival.

## Appendices

**Table 8:** Hourly changes in the median heart rate after initiation of landiolol (unadjusted) Table 8 shows a significant reduction in heart rate (HR, bpm) after the initiation of landiolol (Friedman test: χ²(24) = 155.6, p < 0.001). Post hoc analysis using Dunn’s test revealed that the HR (bpm) was significantly lower at all observation time points than at time 0

Time (h)	Heart Rate (bpm), median (interquartile range)	P-value
0	116 (102–133)	-
1	102 (90–121)	<0.001
2	98 (86–117)	<0.001
3	97 (86–107)	<0.001
4	98 (88–109)	<0.001
5	99 (86–108)	<0.001
6	101 (87–112)	<0.001
7	102 (87–112)	<0.001
8	99 (87–111)	<0.001
9	98 (84–110)	<0.001
10	95 (84–113)	<0.001
11	95 (81–109)	<0.001
12	97 (83–114)	<0.001
13	96 (80–112)	<0.001
14	95 (83–112)	<0.001
15	93 (80–108)	<0.001
16	92 (80–110)	<0.001
17	94 (81–114)	<0.001
18	94 (82–111)	<0.001
19	97 (81–114)	<0.001
20	95 (81–111)	<0.001
21	96 (82–110)	<0.001
22	97 (81–110)	<0.001
23	98 (81–109)	<0.001
24	97 (79–111)	<0.001

**Table 9:** Hourly changes in the median mean arterial pressure after initiation of landiolol (unadjusted) Table 9 shows the changes in mean arterial pressure (MAP, mmHg) after landiolol initiation. An overall increase in MAP was observed following landiolol administration (Friedman test: χ²(24) = 101.3, p < 0.001). Post hoc analysis using Dunn’s test revealed that MAP was significantly higher between 19 and 23 h compared to time 0

Time (h)	MAP (mmHg), median (interquartile range)	P-value
0	70 (61–79)	-
1	68 (61–78)	1
2	67 (59–76)	1
3	69 (60–78)	1
4	72 (63–81)	1
5	71 (62–79)	1
6	72 (62–82)	1
7	68 (61–80)	1
8	71 (62–81)	1
9	70 (61–82)	1
10	74 (62–81)	1
11	72 (62–82)	1
12	72 (62–84)	1
13	72 (64–85)	0.39
14	73 (62–84)	0.14
15	71 (62–82)	1
16	72 (63–83)	0.97
17	70 (64–82)	0.21
18	73 (62–79)	1
19	74 (68–85)	<0.001
20	74 (66–82)	0.033
21	75 (65–84)	0.026
22	75 (65–84)	0.043
23	75 (66–84)	0.01
24	73 (64–83)	0.17
